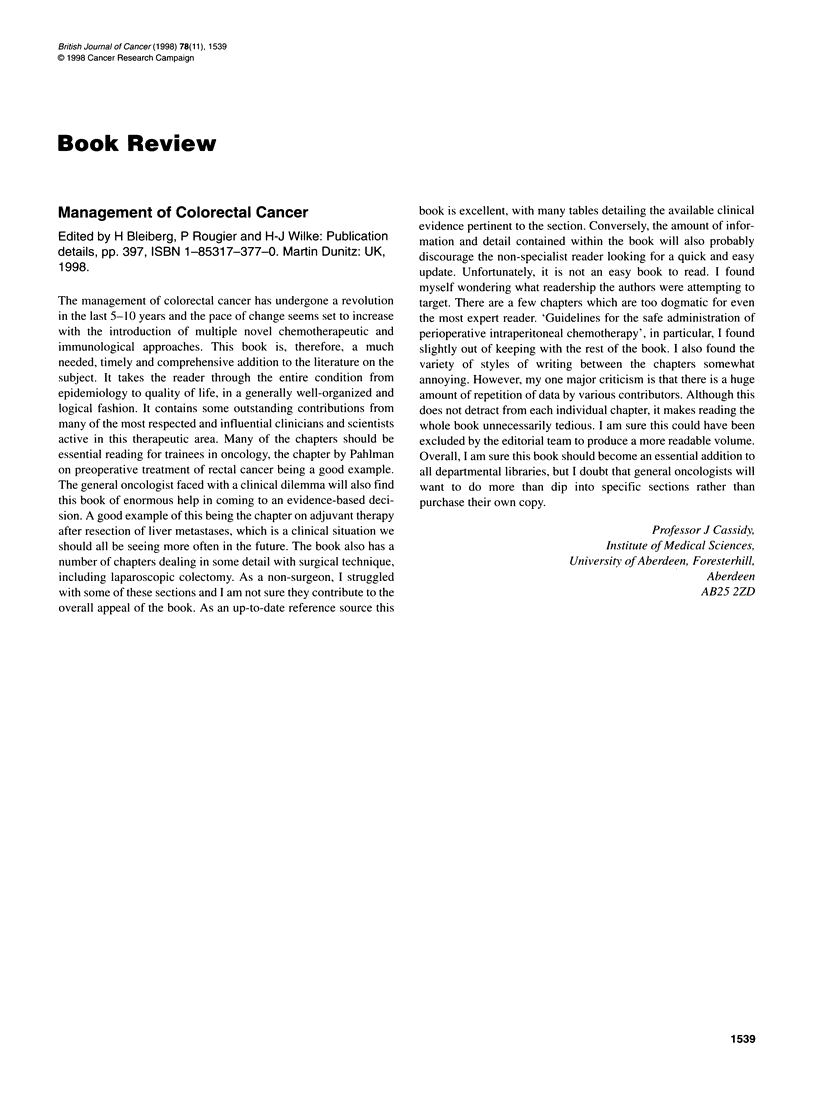# Management of Colorectal Cancer

**Published:** 1998-12

**Authors:** J Cassidy


British Journal of Cancer (1998) 78(11), 1539
© 1998 Cancer Research Campaign

### Book Review

### Management of Colorectal Cancer

Edited by H Bleiberg, P Rougier and H-J Wilke: Publication details, pp. 397, ISBN 1–85317–377–0. Martin Dunitz: UK, 1998.

The management of colorectal cancer has undergone a revolution in the last 5–10 years and the pace of change seems set to increase with the introduction of multiple novel chemotherapeutic and immunological approaches. This book is, therefore, a much needed, timely and comprehensive addition to the literature on the subject. It takes the reader through the entire condition from epidemiology to quality of life, in a generally well-organized and logical fashion. It contains some outstanding contributions from many of the most respected and influential clinicians and scientists active in this therapeutic area. Many of the chapters should be essential reading for trainees in oncology, the chapter by Pahlman on preoperative treatment of rectal cancer being a good example. The general oncologist faced with a clinical dilemma will also find this book of enormous help in coming to an evidence-based decision. A good example of this being the chapter on adjuvant therapy after resection of liver metastases, which is a clinical situation we should all be seeing more often in the future. The book also has a number of chapters dealing in some detail with surgical technique, including laparoscopic colectomy. As a non-surgeon, I struggled with some of these sections and I am not sure they contribute to the overall appeal of the book. As an up-to-date reference source this book is excellent, with many tables detailing the available clinical evidence pertinent to the section. Conversely, the amount of information and detail contained within the book will also probably discourage the non-specialist reader looking for a quick and easy update. Unfortunately, it is not an easy book to read. I found myself wondering what readership the authors were attempting to target. There are a few chapters which are too dogmatic for even the most expert reader. 'Guidelines for the safe administration of perioperative intraperitoneal chemotherapy', in particular, I found slightly out of keeping with the rest of the book. I also found the variety of styles of writing between the chapters somewhat annoying. However, my one major criticism is that there is a huge amount of repetition of data by various contributors. Although this does not detract from each individual chapter, it makes reading the whole book unnecessarily tedious. I am sure this could have been excluded by the editorial team to produce a more readable volume. Overall, I am sure this book should become an essential addition to all departmental libraries, but I doubt that general oncologists will want to do more than dip into specific sections rather than purchase their own copy.

*Professor J Cassidy,*
*Institute of Medical Sciences,*
*University of Aberdeen, Foresterhill,*
*Aberdeen*
*AB25 2ZD*